# Small Intestinal Bacterial Overgrowth and Childhood Malnutrition: A Comprehensive Review of Available Evidence

**DOI:** 10.3390/nu16244319

**Published:** 2024-12-14

**Authors:** Cristina Roxana Mareș, Maria Oana Săsăran, Cristina Oana Mărginean

**Affiliations:** 1 Department of Pediatrics 1, “George Emil Palade” University of Medicine, Pharmacy, Sciences and Technology of Târgu Mureș, Gheorghe Marinescu Street no 38, 540136 Târgu Mureș, Romania; cristina_campean2005@yahoo.com (C.R.M.); marginean.oana@gmail.com (C.O.M.); 2 Department of Pediatrics 3, “George Emil Palade” University of Medicine, Pharmacy, Sciences and Technology of Târgu Mureș, Gheorghe Marinescu Street no 38, 540136 Târgu Mureș, Romania

**Keywords:** small intestinal bacterial overgrowth (SIBO), malnutrition, children, celiac disease, lactose malabsorption, inflammatory bowel disease, cystic fibrosis, short-bowel syndrome

## Abstract

The gut microbiome is essential for children’s normal growth and development, with its formation aligning closely with key stages of growth. Factors like birth method, feeding practices, and antibiotic exposure significantly shape the composition and functionality of the infant gut microbiome. Small intestinal bacterial overgrowth (SIBO) involves an abnormal increase in bacteria within the small intestine. This overgrowth can interfere with digestion, impair nutrient absorption, and lead to both local and systemic inflammation, potentially contributing to malnutrition. In this review, we provide a comprehensive overview of the current understanding of the relationship between SIBO and malnutrition, with a particular focus on the pediatric population. SIBO seems to play an important role in nutrient malabsorption through the gut microbiome imbalance, local inflammation, and disruption of the mucosal intestinal barrier. Additionally, SIBO is more prevalent in digestive disorders linked to malabsorption and malnutrition. Different therapeutic strategies for addressing malnutrition-related SIBO have been proposed. While antibiotics are the primary treatment for SIBO, their effectiveness in promoting weight gain among malnourished children remains uncertain. Hence, future research directed at the impact of microbiome imbalance on nutrient intake and absorption could bring to light new strategies for the effective prevention and treatment of malnutrition.

## 1. Introduction

Malnutrition is an important global concern, affecting approximately 45 million children under the age of five worldwide [1]. Stunting, or low height-for-age, is widespread in resource-limited regions, where it affects more than 30% of children under five [2,3]. Globally, up to 165 million children have been affected by stunting annually, contributing to more than one million deaths under the age of five. Malnutrition, secondary to inadequate nutrition, recurrent infections, and poor sanitation, leads to long-term consequences, such as cognitive delays and increased overall morbidity [4,5]. However, recent studies have demonstrated a relationship between quantitative or qualitative changes in the intestinal microbiota and the development of malnutrition in children from moderate to low-income countries [6,7,8].

The number and diversity of the gastrointestinal microbiota increase from 10^2^ to 10^3^ colony-forming units (CFU) in the duodenum, to 10^8^ in the ileum, and to as many as 10^11^ CFU/mL in the colon, comprising up to 500 different species [9]. The maintenance of this growth pattern of bacterial species seems to be linked to the preservation of a physiological orocecal transit time [10]. Recently, increased research interest led to a better understanding of the impact of these bacteria on nutritional absorption, systemic and local immunity, and other effects of microbial metabolism in the gut [11,12,13]. Many of these studies have investigated the composition of the microbiota rather than the quantity of bacteria in specific parts of the digestive tract.

Small intestine bacterial overgrowth (SIBO) is defined as an increase in the bacterial population of the small bowel over the threshold of 10^5^ CFU/mL, as determined from jejunal fluid aspirate. Since endoscopy is invasive, the depiction of SIBO has been currently based on various types of breath tests, as they are non-invasive diagnostic methods [14].

Growing evidence indicates that SIBO may play a role in the complex pathophysiology of malnutrition. SIBO has been widely associated with a poor socio-economic background and unsanitary living conditions and seems to determine nutrient malabsorption, in terms of protein, fat, and carbohydrate assimilation, through a reduction in the intestinal absorption surface and impairment of the normal functioning of the brush border enzymatic equipment [6,7,8,15,16,17,18]. The complex involvement of SIBO in the pathogenesis and correction of malnutrition is also reflected in the current therapeutic options. First-line malnutrition treatment involves nutritional interventions. However, recent studies indicate that this approach would only reduce stunting by 20% and wasting by 60% in the most affected countries [12,13]. Treatment strategies aimed at modulating the intestinal microbiota involved in malnutrition could improve these outcomes.

On the other hand, intestinal dysbiosis can be secondary to malnutrition and inadequate intake of essential nutrients [19]. Several studies have assessed the influence of diet on the gut microbiota, revealing significant effects on its composition and function [20,21]. For example, a Mediterranean-inspired anti-inflammatory diet made of fruits, vegetables, whole grains, and polyunsaturated fats has been shown to reduce inflammation in Crohn’s disease and normalize the microbiota [21].

In the current review, we summarize the existing literature that correlates the presence of SIBO and malnutrition with a focus on the pediatric population.

## 2. Search Strategy and Selection Criteria

A comprehensive literature search was performed using the PubMed, Scopus, and Web of Science databases for all relevant articles indexed up to October 2024, examining the link between SIBO and malnutrition in both children and adults. The “snowball” method was also applied, where reference lists within articles were reviewed to find additional relevant studies.

The search terms included various combinations of keywords such as “small intestinal bacterial overgrowth”, “SIBO”, “small bowel bacterial overgrowth”, “SBBO”, “microbiota”, “child”, “pediatric”, “malnutrition”, “stunting”, “wasting”, “growth”, “celiac disease”, “inflammatory bowel disease”, “environmental enteric dysfunction”, “cystic fibrosis”, “lactose malabsorption”, “short bowel syndrome”, “antibiotic”, and “probiotic”. Two authors independently screened the titles and abstracts of the initial search results.

**Figure 1:**
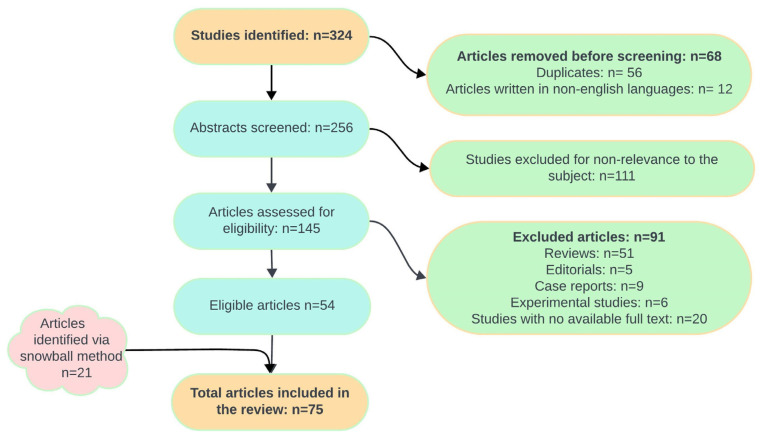
Flowchart of the article selection process.

Research question (PICO) was the main inclusion criteria when selecting the articles: population-based human studies on children and adults with or at risk of malnutrition (population, P); studies including individuals suffering from SIBO, diagnosed via breath tests, jejunal aspirates, or other diagnostic methods (intervention, I); studies including a control group of individuals without SIBO (comparison, C); nutritional status indicators, such as weight-for-age, length-for-age, BMI, and micronutrient deficiencies (outcome, O). Full-text papers, including prospective cohort studies, longitudinal studies, randomized controlled trials, and retrospective cross-sectional studies, were included. Studies that did not meet the research objectives, as well as editorials, letters to the editor, review articles, non-English publications, articles without freely available abstracts, duplicate entries, abstracts, and conference proceedings, were excluded from the review. A flowchart with the schematic representation of the articles selected has been provided in Figure 1. The methodological quality of the studies has been assessed using the STROBE checklist for cohort, case–control, and cross-sectional studies (combined) [22] and the JBI checklist for systematic reviews and meta-analyses [23].

## 3. Results

The database search resulted in 324 articles. After removing duplicates and studies with full texts in languages other than English, 256 studies remained. These were screened based on their titles and abstracts, with 145 found to be relevant to the research question. Of these, 91 were excluded for various reasons. An additional 21 studies were identified using a reference list screening of the included articles. Ultimately, 75 articles met the inclusion and exclusion criteria and were included in the review. More insights into the article selection process are provided in Figure 1.

### 3.1. Normal Small Intestinal Microbiota

The gut microbiome—a complex community of bacteria, viruses, fungi, and archaea—plays an important role in the normal growth and development of children. This includes the metabolism of macro- and micronutrients, vitamin synthesis, hormonal regulation, and the preservation of intestinal mucosal structure and barrier function [24,25,26,27]. Additionally, the gut microbiome influences the development of the immune system and helps protect against colonization by external pathogens and enteric infections [28]. The establishment of the intestinal microbiome coincides with critical periods of child growth. Type of birth, feeding habits, and antibiotic use play an important role in shaping the composition and function of the infant gut microbiome during the first 2–3 years of life [29,30].

Initially, facultative anaerobes are the primary colonizers of the infant gut, followed by obligate anaerobes like *Bifidobacteria*, *Clostridium*, and *Bacteroides*, which produce enzymes involved in digesting human milk [13]. The process of weaning leads to an increase in microbiome diversity, with species like *Fecalibacterium prausnitzii* and *Ruminococcus*, which are involved in complex polysaccharide metabolism, becoming more abundant. By the age of 2–3 years, the gut microbiome begins to resemble that of an adult. This natural maturation of the infant microbiome, primarily influenced by early feeding habits, is a key predictor of child growth [31,32,33].

In healthy adults, the stomach and small intestine contain a small number of bacteria. When present, the bacteria are usually lactobacilli and enterococci, Gram-positive aerobes, or facultative anaerobes, with bacterial counts not exceeding 10³ organisms/mL of jejunal fluid [34].

The terminal ileum serves as a transition zone between the normal flora of the duodenum and jejunum and the microorganisms found in the colon. It predominantly contains aerobic species, unlike the mainly anaerobic flora of the colon. Just before the ileocecal valve, bacterial concentrations range from 10⁵ to 10⁹ organisms/mL, including *Enterobacteriaceae* and strict anaerobes. The colon’s flora primarily consists of anaerobic species such as *Bacteroides*, anaerobic lactobacilli, and *Clostridia*, with concentrations ranging from 10⁹ to 10¹² organisms/mL. In cases where the ileocecal valve is altered, the microorganisms in the terminal ileum increasingly resemble those normally found only in the colon [35].

### 3.2. SIBO Diagnosis in Children

Diagnosing SIBO in the pediatric population is challenging due to the nonspecific symptoms and the difficulties in performing diagnostic tests in children [14,36]. Clinical manifestations of SIBO in children include bloating, abdominal pain, diarrhea, constipation, flatulence, weight loss or failure to thrive, and nutrient deficiencies (e.g., vitamin B_12_ and fat-soluble vitamins) [36].

Given the similarity of these symptoms to conditions such as irritable bowel syndrome (IBS), celiac disease, and lactose intolerance, clinical suspicion alone is insufficient and diagnostic tests are needed. The lactulose breath test (LBT) and glucose breath test (GBT) are the most widely used non-invasive diagnostic tools for SIBO in children. These tests assess hydrogen and methane concentrations in the breath after ingestion of lactulose or glucose, which are digested by the intestinal microbiota. According to the North American consensus, a positive SIBO result is defined as an increase in hydrogen (H₂) of ≥20 parts per million (ppm) from baseline within 90 min of substrate ingestion and/or a methane (CH₄) level of ≥10 ppm at any time during the test [37].

Small intestinal aspiration and culture are the gold standard for SIBO diagnosis. It involves collecting a fluid sample from the small intestine via endoscopy, with bacterial overgrowth defined by more than 10⁵ CFU/mL. While accurate, its routine use in pediatric populations is limited by invasiveness and requirement for sedation [37,38].

### 3.3. Microorganisms Frequently Involved in SIBO and Malnutrition

In SIBO, bacterial overgrowth can arise from the migration of bacteria from either the upper digestive tract or the colon. While it is often caused by a single predominant type of microorganism, it can sometimes involve multiple bacteria [39,40]. Bacteria originating from the colon consist of aerobic and anaerobic organisms. Common Gram-negative bacteria involved in SIBO include *Escherichia coli*, *Proteus*, and *Klebsiella*. In contrast, Gram-positive bacteria like *Enterococcus* and various *Streptococcus* species are less frequent [41]. A recent study using 16S rRNA sequencing found that *Escherichia* and *Klebsiella* accounted for the majority of bacterial overgrowth in SIBO [42]. Anaerobic bacteria such as *Bacteroides, Clostridium*, and *Lactobacillus*, which are normal inhabitants of the colon, can also overgrow in the small intestine, contributing to symptoms like gas production, bloating, and nutrient malabsorption [43]. Methane-producing archaea, particularly *Methanobrevibacter smithii*, may be present in SIBO cases where constipation is a prominent symptom [44]. These organisms convert hydrogen into methane, which can slow intestinal motility and worsen constipation [45].

The research suggests that the intestinal dysfunction seen in severe acute malnutrition (SAM) leads to nutrient malabsorption, potentially driven by a disrupted microbiome [30]. Reduced microbiome maturity or microbiota-for-age Z-score strongly predicts malnutrition in infants [13]. The score was developed in a cohort of healthy infants in Bangladesh and has been validated in both humans and experimental animal models [46]. Moreover, therapeutic feeding for SAM only temporarily restores microbiome maturity, indicating that the microbiome’s regression to an immature state may increase the risk of SAM relapse in infants [46].

### 3.4. Effects of SIBO on Digestion and Absorption

There is increasing evidence supporting the impact of the microbiome on nutritional status, both in animal models and in patients with intestinal dysbiosis. An experiment in which feces from malnourished children were transplanted into germ-free mice demonstrated the development of malnutrition in the animals fed with a typical African diet [6,47]. The beneficial effect of antibiotic treatment on weight recovery in malnourished children is another indicator of the role of gut flora in growth and development [48].

Competition for nutrients between the excessive bacteria overgrowth and the host [49], as well as other SIBO-induced factors (e.g., diarrhea, steatorrhea, carbohydrate malabsorption, protein loss, increased intestinal permeability, intestinal and systemic inflammation), can result in a negative caloric balance, thereby contributing to stunted growth and malnutrition [49].

Intraluminal bacteria overproduction can cause bile acid deconjugation and deficiency, leading to maldigestion and malabsorption of lipids and steatorrhea [11]. Hydroxylated free bile acids (and fatty acids) stimulate the secretion of water and electrolytes, leading to diarrhea.

Deficiencies in fat-soluble vitamins A, D, and E may occur secondary to fat malabsorption, but they are rare and typically remain clinically silent [50]. However, there have been reports of vitamin E deficiency syndromes (neuropathy and T-cell abnormalities) in SIBO [50,51] and a single case report of night blindness caused by vitamin A deficiency [52]. A lack of vitamin B_12_ can be caused by enteric bacteria consumption in the intestinal lumen or by anaerobic organisms inhibiting normal B_12_ absorption [53]. On the other hand, vitamin K and folate levels may be elevated due to bacterial production [54]. While deficiencies in fat-soluble vitamins and vitamin B_12_ have been documented in adults [55,56], no studies have yet explored this issue in children with SIBO.

Deconjugated bile acids such as lithocholic acid can exert a toxic effect on enterocytes, affecting not only the absorption of fats but also carbohydrates and proteins [57]. Carbohydrate malabsorption can also result from the intraluminal breakdown of sugars by bacteria and from the impairment of the disaccharidase activity, as well as of other hydrolases responsible for the digestion of sugars [58]. Lactose intolerance appears to be prevalent in SIBO patients and may play a role in the frequent occurrence of diarrhea. Additionally, the production of toxins by bacteria can directly affect the absorption of proteins and carbohydrates [59,60]. It has been suggested that in SIBO, the microbiota deaminate dietary protein within the GI tract. This leads to a diversion of dietary nitrogen into urea formation, making it unavailable for protein synthesis by the human host [54]. Reduced levels of enterokinases have also been observed in patients with SIBO, which could impair the activation of proteases in pancreatic secretions [61].

Different levels of epithelial inflammation and villous atrophy have been documented in SIBO [62]. A study including aspirate-proven cases of SIBO found villous blunting in 24% of cases and an increase in intraepithelial lymphocytes in 26% of subjects [63]. These alterations play a part in the symptoms linked to SIBO and decrease the amount of intestinal absorptive surface area.

SIBO has been associated with increased intestinal permeability, often referred to as “leaky gut syndrome”. When the gut barrier is compromised, it can lead to the passage of toxins, bacteria, and undigested food particles into the bloodstream [64]. This can trigger immune responses and contribute to systemic and local inflammation, impacting nutrient absorption and overall health [65]. Nutrient absorption and inflammation are likely independent contributors to the pathophysiology of stunted child growth. However, they are also indirectly linked, as inflammation alters the microbiome composition, which in turn affects nutrient absorption and storage [66].

The different pathophysiological mechanisms previously discussed demonstrate how SIBO contributes to malabsorption. In a study investigating SIBO in various pathologies leading to malabsorption (celiac disease, tropical sprue, parasitic infestations, and others), 42% of patients were diagnosed with SIBO [67]. In addition to malabsorption, anorexia, secondary to chronic SIBO symptoms such as bloating, cramps, and diarrhea, also leads to malnutrition [68].

### 3.5. Defense Mechanisms Against SIBO and Predisposing Factors

There are several defense mechanisms against SIBO that influence both the number and types of bacteria in the small intestine [54]. Small intestinal motility, especially the migrating motor complex (MMC), clears bacteria toward the colon every 90–120 min. Impaired motility, as seen in conditions like scleroderma, diabetes, or IBS, allows bacterial stasis and overgrowth [69]. Mucosal immunity, particularly secretory IgA, is also important in preventing bacterial colonization. Therefore, individuals with immunodeficiencies or those on immunosuppressant drugs have an increased risk of developing SIBO [14]. Pancreatic, biliary, and intestinal secretions regulate bacterial populations, while cholestasis or gallstones impair bile flow, promoting SIBO [69]. The acidic pH of the stomach also inhibits bacterial proliferation [70]. Hypochlorhydria from long-term use of proton pump inhibitors or atrophic gastritis (especially in adult patients) compromises this defense, allowing more bacteria to survive and enter the small intestine.

In the following sections, we summarize the data from the literature regarding the relationship between SIBO and other known conditions that cause malabsorption and malnutrition.

### 3.6. SIBO and Environmental Enteric Dysfunction

Environmental enteric dysfunction (EED) is a poorly understood syndrome characterized by inflammation, impaired absorption, and barrier dysfunction in the small intestine. It is common in low- and middle-income countries, affecting approximately 75% of children living in unsanitary conditions [71], and is associated with stunted growth [72,73,74].

Intestinal infections that alter the gut microbiota and lead to EED may play a role in the development of SIBO [72]. Fluid samples from the small intestine of malnourished Indian adults and African children were found to be significantly contaminated with pathogenic bacteria, regardless of the presence of diarrhea [75,76,77].

A study conducted on 90 children in Bangladesh found that SIBO was linked to stunted growth and poor sanitation, though it was not associated with frequent or recent diarrheal disease [15]. The same condition was also linked to intestinal inflammation but not to increased permeability or systemic inflammation.

In a case–control study conducted in Africa on 460 children without gastrointestinal symptoms, 85% of stunted children had a positive SIBO diagnosis from duodenal aspirates. Interestingly, there was an overrepresentation of oral bacteria in both the duodenal aspirates and stool samples of stunted children. The most common genera cultivated from the small intestine were *Streptococcus*, *Neisseria*, *Staphylococcus*, *Rothia*, *Haemophilus*, *Pantoea*, and *Branhamella*, which are generally oral taxa, and their high presence in duodenal fluids is unusual [6].

The same research group further investigated SIBO and EED in a group of 1000 stunted children from Africa. The previous results were confirmed, with the researchers obtaining a high prevalence of SIBO in stunted children (>80%) and an overgrowth of oral bacteria in the small intestine. Additionally, the results indicated low-grade intestinal inflammation in the duodenum of children with SIBO, characterized by elevated levels of duodenal AAT, calprotectin, and various cytokines, including IL-6 and Mcp1 [66].

Another interesting finding of this study concerns the role of SIBO on lipid absorption. In vitro results showed that oral bacteria, primarily *S. salivarius*, isolated from the small intestine, reduce lipid absorption in intestinal epithelial cells. Subsequently, the authors showed that *S. salivarius* decreased intestinal absorption and liver accumulation of dietary fatty acids in a murine model.

These findings suggest that poor sanitation and subsequent EED may increase the risk of developing SIBO. However, none of the studies have clearly identified a pathophysiological mechanism by which such unsanitary conditions lead to the disease. Donowitz et al. [15] proposed a potential mechanism for SIBO development in the context of poor living conditions, suggesting that repeated exposure to elevated levels of liposaccharides found in soil and drinking water may disrupt the migrating motor complex (MMC), causing fecal stasis and thus contributing to SIBO. The studies regarding the relationship between SIBO and EED are included in Table 1.

### 3.7. SIBO and Celiac Disease (CD)

Several studies have noted a significant prevalence of SIBO in individuals with CD, with some suggesting that SIBO may contribute to a poor response to a gluten-free diet (GFD) [78,79,80,81]. One study found that patients with celiac disease and SIBO exhibited signs of malabsorption—such as lower levels of hemoglobin, β-carotene, and albumin, and higher levels of fecal fat—compared to patients with celiac disease who did not have SIBO [78]. However, the findings remain inconsistent. In a meta-analysis that included 14 studies and 742 CD patients, SIBO showed a higher prevalence in patients with CD (18.3%) compared to healthy controls. The prevalence of SIBO was higher in patients unresponsive to dietary treatment compared to those who responded, though the difference was not statistically significant [79]. In this study, no association was found between SIBO and the degree of intestinal damage or serology in patients with CD. The authors could not rule out that SIBO might exacerbate malabsorption in patients with CD, but no statistically significant association was found with markers of malabsorption (hemoglobin, albumin, and fecal fat levels). On the other hand, the meta-analysis showed that almost all (95.6%) SIBO-positive patients with CD who underwent short courses of antibiotic treatment reported improvement of symptoms, which was accompanied by normalization of the breath test. This result was also confirmed in other studies [67,80,81]. Therefore, gut dysbiosis may be the cause of unexplained gastrointestinal symptoms in some patients with CD. As SIBO also causes villous atrophy in approximately 20% of cases [63], the differential diagnosis with CD is challenging, especially in seronegative patients. On the other hand, in celiac patients, SIBO may be a consequence of intestinal epithelial damage and the consequent characteristic dysmotility rather than the cause of the epithelial lesions [82]. The studies regarding the relationship between SIBO and celiac disease are included in Table 2.

### 3.8. Lactose Malabsorption

SIBO is more common in patients with lactase deficiency compared to healthy controls (18.2% vs. 6.7%), even more so in severe lactase deficiency (27.6%) [83]. Additionally, SIBO was detected in 90% of asymptomatic, older individuals with lactose malabsorption, compared to 20% of those without this condition [84]. In one study including 138 patients with post-infectious IBS, 59.4% of patients had secondary lactase deficiency, all of which were diagnosed with SIBO. Sixty patients with lactase deficiency were further randomized into two groups; one received digestive enzymes and spasmolytics, and the other also received probiotics. After two weeks of probiotic treatment, both SIBO and secondary lactose deficiency improved, suggesting that changes in the intestinal microbiota can help relieve symptoms of lactose intolerance [85]. Lactose intolerance and SIBO are directly connected, as SIBO leads to secondary lactose intolerance by damaging mucosal enzymes [83]. The studies regarding the relationship between SIBO and lactose malabsorption are included in Table 3.

### 3.9. SIBO and Inflammatory Bowel Disease (IBD)

A meta-analysis including 11 studies and 1175 IBD patients showed that the prevalence of SIBO in IBD was 22.3%, higher than in the control group [86]. Abdominal symptoms such as bloating, gas, early satiety, and loose stools were more severe in patients with SIBO compared to those without. Crohn’s disease (CrD) patients with SIBO had lower body weight, experienced more frequent bowel movements, were older, had a longer history of IBD, and exhibited prolonged orocecal transit time compared to SIBO-negative patients [86,87,88]. Additionally, a link was identified between the number of surgical procedures and the presence of SIBO (OR = 2.83) [88]. The resection of the ileocecal valve in CrD patients increases the odds of SIBO [86]. Delayed orocecal transit, which can arise from chronic intestinal inflammation, may increase the risk of developing SIBO in IBD patients [89].

SIBO prevalence was found to be higher in patients with CrD—45.2% compared to patients with ulcerative colitis (UC) (17.8%) [87]. UC patients with SIBO exhibit notably higher serum levels of pro-inflammatory cytokines (IL-6, IL-8, and TNF-α), anti-inflammatory cytokines (IL-10), and lipid peroxidases (a marker of oxidative stress), while their levels of reduced glutathione (an antioxidant) are lower compared to UC patients without SIBO [90,91]. The studies regarding the relationship between SIBO and IBD are included in Table 4.

### 3.10. SIBO and Cystic Fibrosis

The prevalence of SIBO in cystic fibrosis (CF) was found to be 30–40% [92,93], with even higher rates in patients with pancreatic insufficiency (56%). SIBO was independently linked to lower BMI and albumin levels [94].

The higher SIBO prevalence in patients with CF [95] is considered to be the consequence of two factors: slowed intestinal transit—possibly due to unabsorbed lipids leading to smooth muscle dysfunction [96,97]; and mucus accumulation, which facilitates bacterial overgrowth [98]. The studies regarding the relationship between SIBO and cystic fibrosis are included in Table 5.

### 3.11. SIBO and Short-Bowel Syndrome

SIBO was diagnosed in 50% of children with short bowel syndrome (SBS) [99]. In SBS, several factors can predispose patients to SIBO, such as the absence of the ileocecal valve, intestinal dilation, stasis due to anastomoses, and substrate accumulation resulting from malabsorption [100,101]. Additionally, children with SBS may be predisposed to SIBO due to the frequent use of antacids and antibiotics. Subjects with SBS and SIBO have a higher risk for bloodstream infections and higher levels of fecal calprotectin compared to SIBO-negative SBS children [102]. The studies regarding the relationship between SIBO and short-bowel syndrome are included in Table 6.

### 3.12. The Treatment of SIBO and Malnutrition

Given the growing evidence of a link between the gut microbiome and child malnutrition, interventions aimed at the gut microbiome have potential as innovative therapies to enhance child growth.

Antibiotics are the fundamental treatment of SIBO, with the most common options being rifaximin, metronidazole, and neomycin. Rifaximin is a nonabsorbable antibiotic that reduces SIBO symptoms and improves gut health [103]. Metronidazole is often used as an alternative or in combination with other antibiotics, with an eradication rate as high as 95% [104]. Neomycin was one of the first antibiotics studied in IBS patients with SIBO; however, the frequent side effects limited its use [105].

The growth-enhancing effects of antibiotics have been recognized and applied in farming for many years. Meta-analyses of antibiotic supplementation in livestock have shown a 16% improvement in growth [106].

Studies have shown a decline in growth patterns among children from developing countries who suffer from recurrent episodes of diarrhea. The connection between diarrhea and malnutrition points to antimicrobials as a primary intervention to disrupt the cycle of enteric infections, diarrhea, and malnutrition [48,107]. This has led the WHO to recommend combining nutritional rehabilitation with antibiotic treatment [108]. This recommendation is backed by a study involving 3000 malnourished children who were treated with antibiotics (amoxicillin, cefdinir, or placebo) [109]. All participants received ready-to-use therapeutic food, yet 15% did not recover from malnutrition. When antibiotics were added to the treatment, the failure rate dropped to less than 11%, mortality decreased, and weight gain increased from 3.1 to 3.9 g/kg/day. However, it remains unclear whether the observed benefits of antibiotics were due to their antimicrobial properties rather than a direct growth-promoting effect.

Metronidazole has also been shown to aid in the recovery from malnutrition in Jamaican children [110]. Patients receiving both a high-calorie diet and metronidazole made significantly greater weight gains than those treated only with diet. Similarly, studies performed in the 1950s showed a stimulatory effect of aureomycin on weight gain [111,112]. More recently, a study performed in Malawi on children with severe malnutrition found that patients treated with cefdinir had a shorter recovery time, greater weight gains, and a reduced mortality rate compared to controls [109]. A systematic review of 10 studies found good evidence supporting the beneficial effects of antibiotics on growth in children from low- and middle-income countries [48]. The growth-promoting effect of antibiotics was more noticeable in terms of weight gain than for height.

The relationship between antibiotic treatment for SIBO and changes in weight is not well-established and can vary depending on several factors. As described before, SIBO often leads to malabsorption of nutrients, which may cause weight loss. In these patients, successful SIBO treatment may lead to weight gain; however, it is not always significant. In one study, underweight patients gained about 0.6 kg after treatment, but no notable weight changes were observed in patients with a higher BMI. However, the follow-up period was short (3 months), making it difficult to assess the long-term effects on weight after SIBO treatment [113].

Prebiotics and probiotics are promising strategies to improve the microbiome associated with child undernutrition; however, definitive evidence of their effectiveness is still lacking. A study on 795 malnourished children from Malawi assessed a combined probiotic/prebiotic intervention but did not show improvements in growth [114]. Conversely, a recent large trial of a combined probiotic/prebiotic treatment aimed at preventing newborn sepsis in India reported a slight increase in infant weight [115]. Furthermore, a small study indicated that *Lactobacillus rhamnosus GG* significantly decreased infections and improved nutritional status in children undergoing treatment for malnutrition [116].

Nutritional support is an important aspect of SIBO treatment, especially for patients experiencing weight loss or deficiencies in vitamins and minerals [117]. Key elements of the treatment include supplementation and maintenance of vitamin B_12_ and fat-soluble vitamins, as well as correcting calcium and magnesium deficiencies. The low-FODMAP diet initially used for IBS includes foods that are less likely to cause fermentation, such as certain vegetables, fruits, and proteins, while limiting grains and dairy products [118]. Patients with SIBO on a low-FODMAP diet experience improvement in symptoms; however, it is not clear if this improvement is due to microbiota changes or just decreased fermentation and subsequent gas production. In severe cases of malnutrition and malabsorption, an elemental diet may be used temporarily to provide essential nutrients while bypassing the need for digestion [119]. In cases where SIBO leads to fat malabsorption, supplementing the diet with medium-chain triglycerides may be needed. Additionally, enzyme supplements, such as pancreatic enzymes, may be necessary to support fat digestion and absorption [119]. Gaffar et al. performed a nutritional intervention on 194 malnourished children from a poor urban community but found no significant difference between SIBO-positive and SIBO-negative groups in response to the diet [120]. However, only 15% of children were SIBO positive, and the follow-up period was short (3 ½ months). The studies regarding growth-promoting effects of antibiotics in malnourished children are provided in Table 7.

## 4. Conclusions

Malnutrition remains an important global issue, especially in low-income countries, where factors such as poor nutrition, infections, and inadequate hygiene contribute significantly to its occurrence. Recent studies suggest that SIBO may play an important role in the complex pathology of malnutrition by affecting digestion, nutrient absorption, and local inflammation. The gastrointestinal microbiota is essential for normal child development, and disruptions in the gut microbiome have been linked to malnutrition. SIBO seems to aggravate the malabsorption syndrome and contribute to malnutrition in patients with CD, IBD, and CF.

Antibiotics are the main treatment for SIBO, with rifaximin and metronidazole proving effective in improving symptoms and nutrient absorption. However, the role of antibiotics in long-term recovery remains debated, and their potential to promote weight gain in malnourished children is still under investigation. The research on the role of diet and probiotic therapy in treating SIBO and malnutrition has yielded conflicting results.

## Figures and Tables

**Table 1:** The relationship between SIBO and environmental enteric dysfunction.

References	Type of Study	Study Group	Gender	Age	Detection Method	Main Outcome	STROBE Compliance
Collard JM et al. [72]	Case–control	109 stunted children	SIBO-positive: 53% females, SIBO-negative: 65% females	2–3 years: 46 SIBO-positive, 6 negative; 4–5 years: 43 SIBO-positive, 14 negative	Duodenal aspirates	SIBO prevalence 85.3%	Partially compliant, no description of study design in title or abstract
Donowitz JR et al. [8]	Cross- sectional	90 Bangladeshi 2-year-olds	51% females	Mean age 24.6 months (24.3–25.1 months)	Breath tests	16.7% of children had SIBO. Logistic regression analysis revealed a significant correlation between the presence of an open sewer and the development of SIBO (OR 4.78, *p* = 0.04). The markers of intestinal inflammation were fecal Reg 1 and fecal calprotectin elevated in SIBO-positive children (*p* < 0.02 and *p* < 0.004). Measures of intestinal permeability and systemic inflammation did not differ between the groups.	Partially compliant, no flow diagram regarding the inclusion of patients
Vonaesch P et al. [6]	Transversal	46 duodenal and 57 gastric samples from stunted children, 404 fecal samples from stunted and non-stunted children living in Bangui, Central African Republic, and in Antananarivo, Madagascar	52% females	Mean age 41 months 2–3 years: 35% 3–4 years: 36% 4–5 years: 29%	DNA extraction and sequencing, culture methods from duodenal aspirates.	The majority of stunted children showed SIBO (100% of samples from Madagascar and 88% from Bangui), dominated by bacteria that normally reside in the oropharyngeal cavity. There was an overrepresentation of oral bacteria in fecal samples of stunted children.	Partially compliant, no description of study design in title or abstract; no objectives clearly stated; no flow diagram regarding the inclusion of patients
Vonaesch P et al. [66]	Cross-sectional	128 duodenal aspirates and 167 gastric aspirates from stunted children, 627 fecal samples from stunted and non-stunted children aged 2–5 from Africa	50% females	2–3 years: 48% 3–4 years: 31% 4–5 years: 20%	DNA extraction and sequencing, culture methods from duodenal aspirates.	SIBO is highly prevalent in stunted children: 88.1%. SIBO is characterized by an overgrowth of oral bacteria. The duodenal bacteria impair lipid absorption in cultured enterocytes and mice.	Partially compliant, no flow diagram regarding the inclusion of patients

**Table 2:** The relationship between SIBO and celiac disease.

References	Type of Study	Study Group	Gender	Age	Detection Method	Main Outcome	STROBE Compliance/JBI Checklist for Systematic Reviews and Meta-Analysis
Shah A et al. [79]	Meta-analysis	14 studies, 742 CD patients and 178 controls	Not available	Not available	Breath tests and duodenal aspirates	The pooled prevalence of SIBO in CD patients was 18.3%. Using BTs, SIBO prevalence was 20.8%, and culture-based methods yielded a prevalence of 12.6%. SIBO prevalence in non-responsive CD patients is not statistically higher than in responsive CD patients. Antibiotic treatment of SIBO-positive patients resulted in symptom improvement in 95.6% of cases.	JBI checklist compliant
Rubio-Tapia A et al. [78]	Cross-sectional	149 patients with biopsy-confirmed CD	77% females	Mean age 55 years (22–94 years)	Quantitative culture of duodenal aspirate	9.3% prevalence of SIBO in CD patients, 11% SIBO prevalence in non-responsive CD patients. SIBO patients had lower hemoglobin, β-carotene, and albumin levels and a higher amount of fat in stool.	Partially compliant with STROBE criteria: no data on the location of the study, no flow diagram regarding the inclusion of patients, no limitations of the study
Ghoshal U et al. [67]	Case–control	50 patients with various causes of malabsorption, out of which 5 with CD	62% males	Mean age 44 ± 8.5 years	Duodenal aspirates	42% of patients with malabsorption syndrome and 2/5 CD patients with SIBO. Bacteria were more often sensitive to quinolones.	Partially compliant with STROBE criteria: no description of study design in the title or abstract, no specific objectives, no flow chart, no limitations of the study
Tursi A et al. [80]	Experimental	15 CD patients	66% females	Mean age 36.5 years, range 24–59 years	Breath tests	10/15 patients were SIBO positive. All SIBO-positive patients → improvement of symptoms after Rifaximin treatment.	Partially compliant with STROBE criteria: no description of study design in the title or abstract, no data on the location of the study, no flow chart, no limitations of the study
Rana SV et al. [81]	Case–control	87 CD patients and 87 matched controls	56% males	The mean (± SD) age for male patients was 26.3 ± 16.3 years (range 14–59 years), and for female patients was 28.4 ± 15.6 years (range 16–58 years)	Breath tests	20.7% of CD patients and none of the controls were SIBO-positive	Partially compliant with STROBE criteria: no description of study design in the title or abstract, no flow chart, no limitations of the study

**Table 3:** The relationship between SIBO and lactose malabsorption.

References	Type of Study	Study Group	Gender	Age	Detection Method	Main Outcome	STROBE Compliance
Ruchkina IN et al. [85]	Randomized control trial	60 patients with secondary lactase deficiency were randomized into 2 groups, one receiving digestive enzymes and spasmolytics and one also receiving probiotics	81.2% females	Mean age 33.9 ± 9.1 years	Breath tests	All patients with secondary lactase deficiency were diagnosed with SIBO. Breath test normalized in 70.8% of patients with SIBO after probiotic treatment.	Partially compliant, no description of study design in title or abstract;
Jo IH et al. [83]	Case–control	88 patients with digestive symptoms after dairy consumption, 30 controls	54.5% females	Mean age 50.8 years	Breath tests, endoscopy, lactose intolerance quick test	18.2% of patients were SIBO positive, 27.6% of patients with severe lactose deficiency were SIBO positive	Partially compliant, no description of study design in title or abstract;
Almeida JA et al. [84]	Case–control	20 asymptomatic elderly volunteers, 20 asymptomatic younger subjects as controls	55% females	Median age 79 years, range 70–94 years	Breath tests, mannitol absorption test, duodenal aspirates	Lactose malabsorption in 50% of elderly volunteers and 5% of younger subjects, 9/10 elderly subjects with lactose malabsorption were SIBO positive	Partially compliant, no description of study design in title or abstract; no limitations of the study

**Table 4:** The relationship between SIBO and IBD.

Reference	Type of Study	Study Group	Gender	Age	Detection Method	Main Outcome	STROBE Compliance/JBI Checklist for Systematic Reviews and Meta-Analysis
Shah A et al. [86]	Meta-analysis	11 studies, including 1175 patients with IBD and 407 controls	Not available	Not available	Breath tests	SIBO prevalence of 22.3% in IBD patients	JBI checklist compliant
Gandhi A et al. [90]	Meta-analysis	7 studies, including 626 IBD patients and 497 controls	Not available	Not available	Breath tests	The prevalence of methane-positive SIBO was 7.4% in IBD patients compared to 23.5% in controls and in 5.4% of CrD patients compared to 20.2% in UC patients.	JBI checklist compliant
Rana SV et al. [87]	Case–control	137 IBD patients (95 UC and 42 CrD) and 115 healthy controls	59% males	Mean age 44.5 years, range 20–65 years	Breath tests	SIBO prevalence is higher in IBD patients (26.3%) than in controls and in CrD patients (45.2%) than in UC patients (17.8%). OCTT is significantly higher in IBD patients than controls	Partially compliant with STROBE criteria: no description of study design in the title or abstract, no flow chart, no limitations of the study
Greco A et al. [88]	Cross-sectional	68 CrD patients	61% males	Mean age 49.3 ± 12.8 years	Breath tests	SIBO prevalence was 26.5%. Breath test normalized in 13/15 patients treated with antibiotics and probiotics	Partially compliant with STROBE criteria: no description of study design in title or abstract, no flow chart
Rana SV et al. [91]	Case–control	120 UC patients, 125 controls	61% males	Mean ± SD age of 45.6 ± 17.5 years	Breath tests	SIBO prevalence is higher in UC patients (15%). There was a significant correlation between SIBO and IL-6, IL-8, TNF-α, and IL-10 and lipid peroxidases.	Partially compliant with STROBE criteria: no description of study design in the title or abstract, no flow chart, no limitations of the study

**Table 5:** The relationship between SIBO and cystic fibrosis.

Reference	Type of Study	Study Group	Gender	Age	Detection Method	Main Outcome	STROBE Compliance
Furnari M et al. [92]	Case–control	79 CF patients and 25 SIBO-positive patients were further randomized into 2 groups, one receiving rifaximin and one receiving no treatment	50% males	Median age 19.6 years; range 9.2–36.9	Breath tests	31.6% of CF patients were SIBO-positive, with a significant correlation with lower BMI and serum albumin levels. The eradication rate of SIBO was 90% in the rifaximin group and 33% in the control group.	Strobe compliant
Lisowska A et al. [93]	Case–control	25 CF patients and 30 controls	Not available	Median age 9.4 years, range 5–16	Breath tests	40% CF patients were SIBO positive Fecal calprotectin concentrations significantly higher in CF patients	Partially compliant with STROBE criteria: no description of study design in title or abstract, no flow chart
Fridge JL et al. [94]	Case–control	25 CF patients with pancreatic insufficiency, 25 controls	60% males	Mean age 17 years, range 6–46 years	Breath tests	56% CF patients were SIBO positive	Partially compliant with STROBE criteria: no description of study design in the title or abstract, no flow chart, no limitations of the study

**Table 6:** The relationship between SIBO and short-bowel syndrome.

Reference	Type of Study	Study Group	Gender	Age	Detection Method	Main Outcome	STROBE Compliance
Seddik TB et al. [99]	Retrospective	35 children with SBS	54% males	<2 years 26% 2–4 years 43% 5–9 years 21% >10 years 10%	Clinical symptoms	50% of SBS patients positive for SIBO	Partially compliant: no flow chart
Phyo LY et al. [101]	Case–control	13 patients with SBS, 7 controls	54% females	Mean age 6.4 years; range 1–10 years	16S RNA gene sequencing	SBS patients treated with antibiotics for SIBO prophylaxis had an increase in *Firmicutes*, *Proteobacteria*, and *Escherichia coli* species.	Partially compliant: no description of study design in the title or abstract, no flow chart
Cole CR et al. [102]	Case–control	10 infants with SBS caused by necrotizing enterocolitis, 5 healthy controls	60% males	Mean age 7.2 months; range 4.2–15.4 months	Breath tests	50% of SBS patients positive for SIBO; SIBO increased the odds of bloodstream infections (>7-fold, *p* = 0.009). Calprotectin levels are higher in children with SBS and SIBO (*p* < 0.05).	Partially compliant: no description of the study design in the title or abstract, no flow chart, no limitations of the study

**Table 7:** Growth-promoting effects of antibiotics.

Reference	Country	Number of Patients	Gender	Age	Treatment	Results	STROBE Compliance
Mackay et al., 1995 [112]	Jamaica	955	52% males	4–16 years	Aureomycin	Slight positive effect on growth for aureomycin, no effect for vitamin B12	Not compliant: no abstract, no description of study design, no flow chart, no objectives, no description of statistical methods
Guzman et al., 1958 [111]	Guatemala	332	54% males	6–12 years	Aureomycin or Penicillin	Aureomycin initially stimulated growth, increasing both weight and height. The increase in weight gain was more pronounced than in height gain. In contrast, Penicillin appeared to inhibit both weight and height gains.	Not compliant: no abstract, no description of study design, no flow chart, limited description of statistical methods
Heikens et al., 1993 [110]	Jamaica	81	Not available	Mean age 1.2 years	Metronidazole	Children receiving both high energy supplements (HES) and metronidazole made significantly greater gains than those who only received HES (weight, *p* = 0.02; length, *p* = 0.0002; and BMI, *p* = 0.0001	Partially compliant: no description of study design, no flow chart
Trehan et al., 2013 [109]	Malawi	2767	Not available	6–59 months	Amoxicillin, Cefdinir	Amoxicillin reduced the mortality rate by 35.6%, while cefdinir achieved a 44.3% reduction. Children who received cefdinir showed the most significant increases in weight and mid-upper-arm circumference.	Partially compliant: no flow chart, no limitations of the study
